# Very High Fascioliasis Intensities in Schoolchildren from Nile Delta Governorates, Egypt: The Old World Highest Burdens Found in Lowlands

**DOI:** 10.3390/pathogens10091210

**Published:** 2021-09-17

**Authors:** M. Victoria Periago, M. Adela Valero, Patricio Artigas, Verónica H. Agramunt, M. Dolores Bargues, Filippo Curtale, Santiago Mas-Coma

**Affiliations:** 1 Departamento de Parasitología, Facultad de Farmacia, Universidad de Valencia, Av. Vicent Andres Estelles s/n, 46100 Valencia, Spain; vperiago@mundosano.org (M.V.P.); Madela.Valero@uv.es (M.A.V.); Patricio.Artigas@uv.es (P.A.); Veronica.Hernandez@uv.es (V.H.A.); 2 U.O.C. Rapporti internazionali, con le Regioni e Gestione del Ciclo di Progetto, Istituto Nazionale per la Promozione della Salute delle Popolazioni Migranti e il Contrasto delle Malattie della Povertà, 00153 Roma, Italy; filippocurtale@hotmail.com

**Keywords:** human fascioliasis, children, coprology, high intensities, gender and age, geographical distribution, Alexandria and Behera governorates, Nile Delta, Egypt

## Abstract

Quantitative coprological analyses of children were performed in Alexandria and Behera governorates, Egypt, to ascertain whether individual intensities in the Nile Delta lowlands reach high levels as those known in hyperendemic highland areas of Latin America. Analyses focused on subjects presenting intensities higher than 400 eggs per gram of faeces (epg), the high burden cut-off according to WHO classification. A total of 96 children were found to shed between 408 and 2304 epg, with arithmetic and geometric means of 699.5 and 629.07 epg, respectively. Intensities found are the highest hitherto recorded in Egypt, and also in the whole Old World. A total of 38 (39.6%) were males and 58 (60.4%) were females, with high intensities according to gender following a negative binomial distribution. The high burden distribution shows a peak in the 7–10 year-old children group, more precocious in females than males. Results showed high burdens in winter to be remarkably higher than those known in summer. The fascioliasis scenario in Egyptian lowlands shows similarities to highlands of Bolivia and Peru. Diagnostic methods, pathogenicity and morbidity in high burdens should be considered. The need for an appropriate quantitative assessment of heavy infected children to avoid post-treatment colic episodes is highlighted.

## 1. Introduction

Fascioliasis is a disease caused by two trematode species: *Fasciola hepatica* and *F. gigantica*. Whereas in Europe, the Americas and Oceania only *F. hepatica* is present, the distribution of both species overlaps in many areas of Africa and Asia [1]. This disease is transmitted by freshwater lymnaeid snails [2,3] and infects mainly herbivore mammals, among which sheep, cattle, goats, buffaloes and equines are the most important reservoirs. Humans are infected by many different sources, which vary according to countries, diet and traditions. Sources mainly include several vegetables, drinking of natural freshwater or combinations of both, transporting the infective stage of metacercaria [4].

The amphibious characteristics and marked environment dependence of the lymnaeid snail vectors and the management, movements and transport of livestock underlie the great impact of climate change and global change on this disease, respectively [5,6]. These changes are linked to the increasing number of human fascioliasis reports and higher infection risk not only in endemic areas of low-income countries but also in developed countries [7].

Fascioliasis is a disease whose pathogenicity and clinical characteristics was traditionally linked to its acute phase [8]. However, the finding of very high prevalences and intensities in human endemic areas of Bolivia [9,10], Peru [11,12] and Egypt [13] has revealed long-term infections and repeated reinfections.

Fascioliasis has a great impact in its chronic and advanced chronic stage. Fascioliasis chronicity may last for many years in humans [14], cause severe pathology [15,16], give rise to sequelae even in treated subjects [17], leave handicapped subjects behind and even cause the death of the patients [17]. Additional studies demonstrated the complex immunomodulation effect at the acute phase [18]. In the chronic phase, T cells are induced to enter an anergic state characterised by decreased cytokine responses and reduced proliferative activity [19] and may explain why these cells become hyporesponsive to antigen stimulation in the chronic phase. The immune suppressive effect during the long chronic phase underlies the absence of premunition [20] and may enhance the health problematics in the highly frequent coinfection cases with other parasitic diseases [21]. Moreover, reinfection during the chronic phase appears related to an increase in egg output [22].

The aforementioned aspects led the World Health Organisation (WHO) to include fascioliasis in the group of foodborne trematodiases within the priority list of neglected tropical diseases (NTDs) [23] and in the most recent efforts for worldwide sustainability in the prevention and control activities against these diseases [24]. In 2007, after the agreement of the donation of Egaten^®^ (Novartis Pharma AG, Basel, Switzerland) (triclabendazole for human use) [25], WHO launched a worldwide initiative against human fascioliasis. Initial steps included different strategies according to four selected human endemic countries [26]. In Vietnam, treatment of infected subjects was performed in hospitals by passive detection after appropriate radiophonic diffusion. Preventive chemotherapy included yearly campaigns of mono-dose mass treatments in the permanent transmission area of the Northern Altiplano of Bolivia and the seasonal transmission area of the Cajamarca valley in Peru [27,28]. In Egypt, subjects were treated after active detection by qualitative coprological diagnosis with the fast and simple Kato-Katz technique [29,30], by the survey teams of the Ministry of Health and Population (MoHP) located throughout the endemic rural areas and already working on another freshwater snail-borne trematodiasis such as schistosomiasis [31]. Moreover, a multidisciplinary One Health intervention is underway to complement preventive chemotherapy in Bolivia, to decrease infection and re-infection risks in between the annual treatment campaigns [4,6,32,33].

In helminthiases, the intensity of infection, measured as eggs per gram of faeces (epg), is considered a reliable indicator of infection risk and a useful predictor of later morbidity [34]. In human hyperendemic areas, very high fascioliasis prevalences have been found in surveys focused on both schoolchildren and entire populations. In the Northern Bolivian Altiplano, prevalences of up to 72% and 100% have been recorded in certain localities according to coprological [10,35] and immunological surveys [9,36], respectively. These prevalences overlap with very high individual infection intensities, such as 5064 epg in Bolivian children [10] and more recently up to 8088 epg in a nine-year-old girl and also an eight-year-old boy also from the Northern Altiplano area [1]. In Peru, human prevalence also appear to be high in several areas, such as the Altiplano of Puno and the Cajamarca valley, where burdens were up to 2496 epg [11] and 864 epg [12], respectively.

In South America, such high intensities have been recorded in Andean highlands, where the high transmission rates are related to strategies developed by the liver fluke and the lymnaeid snail vectors to adapt to the extreme environmental characteristics of very high altitude areas. These adaptation strategies favour disease transmission, including a longer cercarial shedding period, a greater cercarial production per snail, a longer survival of infected snails [37] and a smaller development of the fluke uterus [38].

**Figure 1:**
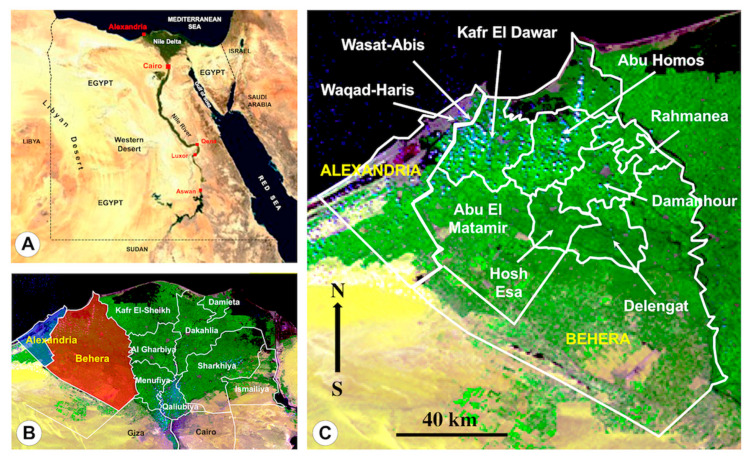
Maps showing endemic areas studied: (**A**) General map of Egypt showing the northern Lower Egypt including the large Nile Delta and the southern Upper Egypt including the localities of Qena and Luxor; (**B**) map showing the governorates of the Nile Delta, including the western governorates of Alexandria and Behera; (**C**) map of the governorates of Alexandria and Behera, including the districts where human samples were collected. Background for A from composed satellite map of Africa orthographic projection by NASA (full resolution of 1624 × 1824 pixels; public domain) via Wikimedia Commons. Backgrounds for B and C from Probe-V satellite by European Space Agency (ESA: https://www.esa.int/ESA_Multimedia/Keywords/Location/Egypt/(sortBy)/view_count/(result_type)/images), high resolution 944.58 kB image accessed on 30 June 2021. Original S. Mas-Coma.

The question is posed on whether such high burdens and consequent individual high morbidities may be caused by fascioliasis in lowlands. The human hyperendemic area of the Nile Delta in Egypt was selected for this assessment (Figure 1). A drastic increase in the number of human fascioliasis cases has been detected in this wide Egyptian region since 1980 [31,39]. Human infection has been reported in different governorates [40], i.e., in Alexandria, Behera, Cairo, Dakahlia, Kafr El-Sheikh, Qalyoubia, Menoufia and Sharkia. A total of 830,000 people were estimated to be infected by liver flukes in the whole Nile Delta region [41]. Coprological surveys in the Behera governorate have shown very high prevalences in total population, ranging between 5.2% and 19.0% depending on localities (mean: 12.8%) [13]. These prevalences were the highest recorded in Egypt, suggesting that previous WHO analyses may have underestimated the real situation, and furnished a picture similar to that known in Andean countries. An impact on anaemia in children infected with liver flukes in Egypt has been highlighted [31,42]. Qualitative diagnostic and selective treatment campaigns by administration of triclabendazole were implemented since 1998 [43]. The analysis of 21,477 subjects, namely primary schoolchildren covered by a control programme, showed prevalence and intensity among 932 positive cases to be higher in girls than boys [44].

However, intensities between 24 and 432 epg (arithmetic mean: 72 epg; geometric mean: 51 epg) found in Egypt [13] were pronouncedly lower than those known in Bolivia or Peru. This was initially related to normal transmission rates in lowlands, which are lower than in highlands [37]. Nevertheless, intensity results obtained in that study were not considered conclusive, since the surveys were performed only in June [13], whereas data available suggest a seasonality in fascioliasis transmission in the Nile Delta [45], similar to other Mediterranean endemic agricultural areas [46].

The present study includes an analysis of the high intensity cases detected in the governorates of Alexandria and Behera by only focusing on positive cases with intensities higher than 400 epg, to (i) show that infection intensities may also reach high levels in the Egyptian Nile Delta, i.e., in lowlands of countries of the Old World, and to (ii) compare them with the characteristics of the high infection intensities described in the highlands of South America.

## 2. Results

From a total of 6657 children, the previous qualitative diagnostic analyses furnished Kato-Katz slides labelled as presenting several eggs in a total of 362 children. These slides were microscopically re-checked for the exact egg counting. Stool samples from 96 children demonstrated to present more than 400 epg. Egg counts showed a range between 408 and 2304 epg, with arithmetic mean and geometric mean of 699.5 and 629.07 epg, respectively. All these high intensity cases originated from surveys performed in the February–March period during the years 1998–2007.

The distribution of these children presenting high intensities according to governorates and districts is shown in Table 1. Significant differences were detected in the percentages of children with epg >400 between districts, although only in the governorate of Behera (*p* = 0.0031) but not in governorate of Alexandria (*p* = 0.9467). The Behera district of Hosh Esa is the one where a higher percentage of high intensity cases were detected, followed by Kafr El Dawar, Abu Homos, Abu El Matamir and Delengat, all at a very similar rate (Figure 1). Even though, the comparison of the mean intensities per district shows that they are higher in Abu Homos and Hosh Esa. In Alexandria governorate, both districts (Figure 1) presented the same number of high intensity cases (Table 1). However, significant differences were not detected between districts.

**Figure 2:**
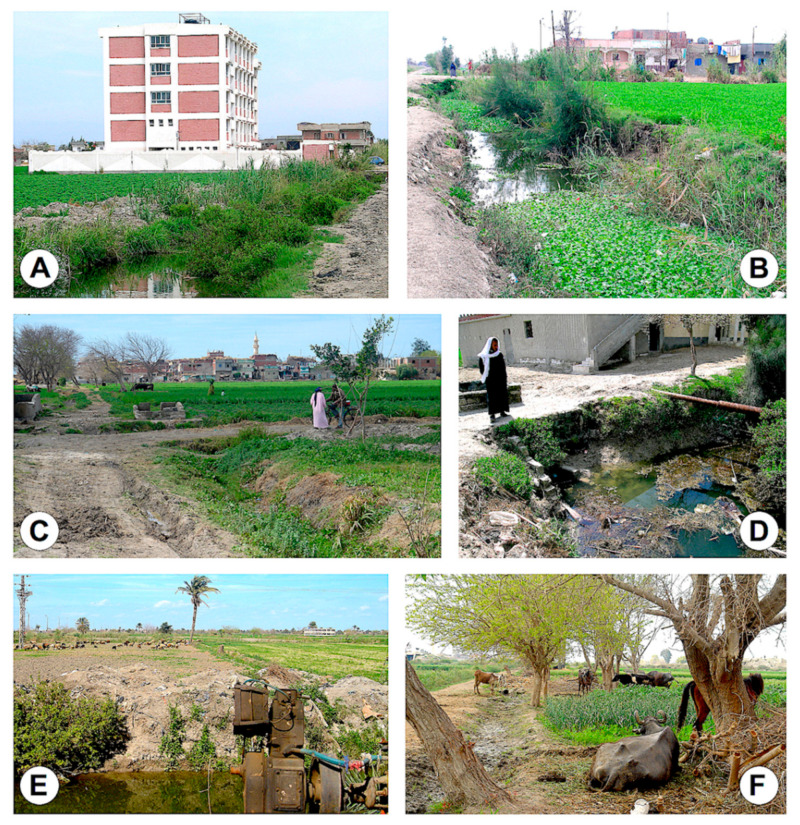
Fascioliasis transmission foci inhabited by lymnaeid snail vectors in the Alexandria governorate: (**A**) Water canal between cultivated field and road besides school of children in Abis 8 Village 1, Wasat district; (**B**) similar canal with abundant water hyacinth close to human dwellings in Village 11 of Abis 8, Wasat-El Raml district; (**C**) irrigation canal in front of city suburb in Village 1 of Abis 8, Wasat-El Raml district; (**D**) freshwater collection besides human dwellings in El Missiry, Waqad-Haris district; (**E**) sheep herds are numerous in Village 4 of Abis 8, Wasat-El Raml district; (**F**) cattle, buffaloes, donkeys and horses are usually present around Village 4 of Abis 8, Wasat-El Raml district. (**A**,**B**,**D**): Photographs by P. Artigas; (**C**,**E**,**F**): Photographs by S. Mas-Coma.

**Figure 3:**
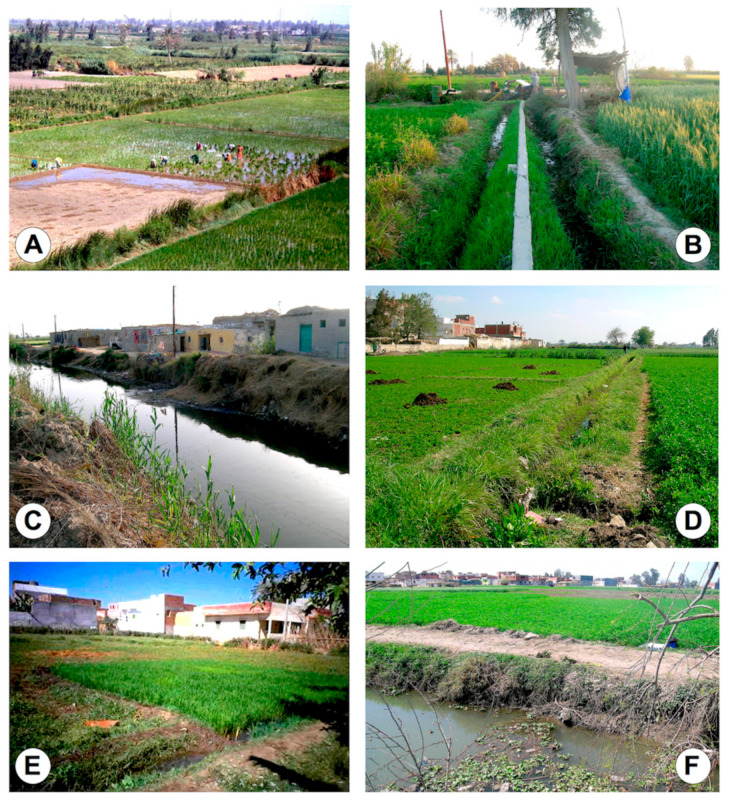
Fascioliasis transmission foci inhabited by lymnaeid snail vectors in the Behera governorate: (**A**) Rice fields observed from the fourth floor of school of children in El Kazza, Hosh Esa district; (**B**) irrigation canals filled by water pump in El Kazza, Hosh Esa district; (**C**) large secondary canal besides human dwellings in El Kazza, Hosh Esa district; (**D**) small canal for irrigation of cultivated field in the way for livestock manure fertilisation besides village in Zuhra, Kafr El Dawar district; (**E**) small canals for rice field irrigation close to human dwellings in Bolin El Aaly, Kafr El Dawar district; (**F**) wider canal with water hyacinth with village in the background in Tiba, Delengate district. (**A**,**E**): Photographs by S. Mas-Coma; (**B**–**D**,**F**): Photographs by P. Artigas.

All districts in both Alexandria and Behera governorates are large irrigated flatlands presenting similar fascioliasis transmission foci (Figure 2 and Figure 3), including from small irrigation canals sometimes artificially filled by water pumps (Figure 3B) and typically inhabited by small, more amphibious lymnaeid snails transmitting *F. hepatica*, up to wider and deeper canals, usually presenting water surface coverage by the typical water hyacinth (Figure 2B and Figure 3F), usually inhabited by bigger, more aquatic lymnaeid snails transmitting *F. gigantica*. Besides the aforementioned transmission foci, there are also crucial epidemiological aspects in these areas that are important to note: (i) the presence of schools (Figure 2A and Figure 3A), (ii) the proximity to human dwellings (Figure 2B,D and Figure 3C,E) and (iii) also the proximity to city suburbs (Figure 2C), (iv) the presence of main livestock reservoir species such as sheep, cattle, buffaloes, donkeys and sometimes even horses, all around (Figure 2E,F), and (v) the surrounding cultivated fields frequently fertilised with livestock manure (Figure 3D).

**Figure 4:**
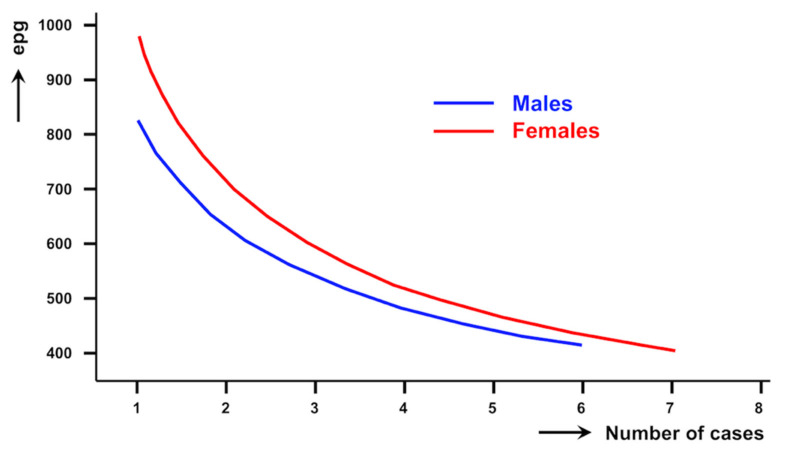
Curves of intensities found in fascioliasis-infected children from the Nile Delta region, Egypt, according to sex. epg = eggs per gram of faeces.

Detailed egg counts detected in the Kato-Katz slides of the aforementioned 96 highly infected children, including distribution by gender and age, are noted in Table 2. When arranged according to groups of egg counts, the distribution of the number of fascioliasis-infected children presenting high epg counts follows a negative binomial distribution (Table 3; curve not shown but see Figure 4).

High intensities were detected in both sexes. With regard to the intensity distribution according to gender, a total of 38 (39.6%) were males and 58 (60.4%) were females. Although no significant differences were found (*p* = 0.1043), girls appear to predominate in all groups of egg counts (Table 3). The intensities according to gender also follow a negative binomial distribution (Figure 4).

**Figure 5:**
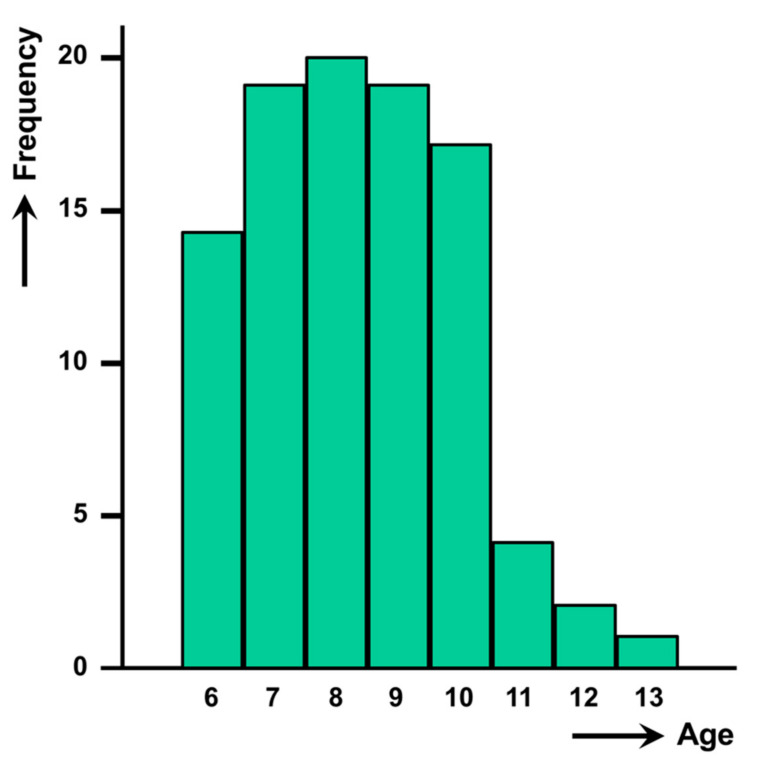
Distribution of the number of fascioliasis-infected children presenting high intensities in the Nile Delta region, Egypt, according to age.

The high intensities were detected in the 6–13 year old groups. The detailed individual distribution of intensities according to age is noted in Table 2 (*p* < 0.0001). Significant differences were detected in % of children with epg >400 according to years of age. The 7–10 year-old groups are those where the higher percentages were found (Table 2). This peak at the 7–10 year-old group clearly appears when analysing the curve of distribution of the number of children studied (Figure 5).

**Figure 6:**
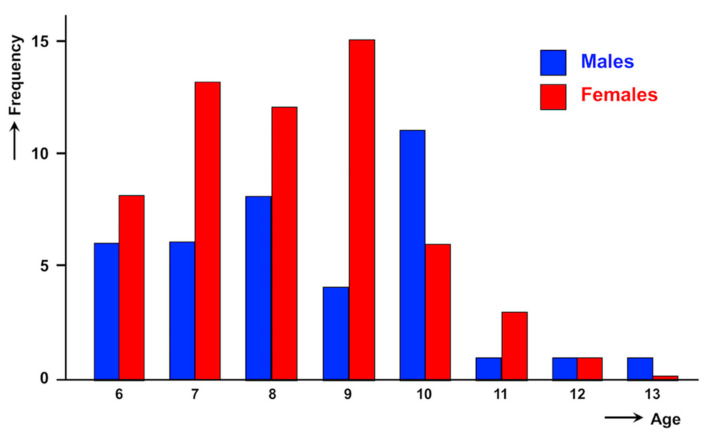
Distribution of the number of fascioliasis-infected boys and girls presenting high intensities in the Nile Delta region, Egypt, according to age.

However, no significant differences were detected in epg values when comparing between age groups (*p* = 0.320). The highest intensities detected are mostly in the 9-year old group (Table 2). Moreover, when the curve is divided between males and females, results suggest that the peak in age is more precocious in females than in males (Figure 6).

A total of 24 of the 96 children highly infected by liver flukes (25.0%) were also shedding eggs of *Schistosoma mansoni* in their faeces. Significant differences were detected in the % of co-infected and the % of non-coinfected children (*p* < 0.0001).

## 3. Discussion

### 3.1. Characterisation of the High Intensities

In Egypt, prevalences found in surveys in districts of the Behera governorate indicate situations to be catalogued as either hyper- or mesoendemic [13]. However, although the fascioliasis intensities detected in these areas were high when compared to those found in subjects sporadically infected in animal endemic areas, as in Europe [8], they appeared to be relatively low considering the high prevalences found [13]. The majority (85%) of subjects infected was shedding 100 epg or less and the maximum egg output was of 432 epg. These egg counts and the corresponding arithmetic and geometric epg means of 72 and 51 epg, respectively, found in these surveys were far from those known in the human hyperendemic areas of South America. In the Northern Bolivian Altiplano, the highest maximum ranges found were 5064 epg in schoolchildren and 4440 epg in the community surveys, with arithmetic and geometric means of 446 and 191 epg when including all the subjects analysed, 419 and 185 epg (24–5064 epg) when restricting to schoolchildren, and 660 and 250 epg (24–4440 epg) in all the communities surveyed. The highest arithmetic and geometric means were 705 and 323 epg in schoolchildren, and 1345 and 678 epg in the community surveys, respectively [10,47]. In the Peruvian Altiplano of Puno, burden results included intensities of up to 2496 epg, with an arithmetic mean of 196–350 epg (mean: 279 epg) and a geometric mean of 96–152 epg (123 epg) [11].

The intensities reported in the present study are the highest hitherto recorded not only in Egypt, but also in the whole Old World. So far, fascioliasis intensities have never been the aim of detailed studies in human surveys in Egypt by other authors [40]. In Egypt, the Kato-Katz technique, despite being a quantitative method, is widely used for mere qualitative purposes. In the few papers in which egg counts were mentioned, ranges of low or moderate levels of less than 250 epg were always noted [42,48,49,50]. However, sporadic cases presenting very high egg counts, such as 936 and 2016 epg, had been previously reported [31,39].

The results obtained in the present study show that intensities remarkably higher than those previously detected [13] are reached in the human hyperendemic areas of the governorates of Alexandria and Behera. However, the intensities found in Egyptian schoolchildren appear to be somewhat lower than those known in Bolivia and Peru, although the cases presenting more than 1000 epg and the maximums of more than 2000 epg (two females with 2304 and 2040 epg, respectively) clearly resemble the epidemiological situations in Andean countries [11,47,51]. The high intensities detected in schoolchildren indicate that there are places in the Alexandria and Behera areas surveyed presenting a very high human contamination risk. This is worth noting because the Egyptian hyperendemic areas are flat lowlands near to sea level in which the liver fluke and the lymnaeid vectors should a priori not have developed particular life cycle strategies to enhance transmission rates such as those observed in very high altitude areas [37].

### 3.2. Relationship with Geographical Distribution

Only slight differences appear when comparing the number of children presenting high intensities according to districts. Indeed, the highest numbers concern the districts where the intensities were re-checked in a higher number of children, such as the districts of Kafr El Dawar and Hosh Esa. The lowest number in the Damanhour should be related to the large urbanisation of this district which includes the capital of the Behera governorate.

This geographical picture of fascioliasis according to districts speaks about the homogeneous epidemiological scenario offered by the flatlands of both governorates of Alexandria and Behera (Figure 2 and Figure 3), concerning:

- The man-made irrigation systems of the wide plant culture fields frequently using livestock manure for fertilisation;
- The overall distribution of livestock reservoir species among which the absence of the pig and the addition of the buffalo should be mainly highlighted when comparing to Latin America endemic areas.
- The coexistence of small-superficial and large-deeper irrigation canals allowing for the wide distribution of smaller, more amphibious lymnaeid species of the *Galba*/*Fossaria* group assuring *F. hepatica* transmission, and bigger, more aquatic lymnaeid species of the *Radix* group assuring *F. gigantica* transmission, respectively, as well as together assuring the viability of intermediate hybrid forms [52].

Similar to other freshwater-borne parasitic diseases, fascioliasis presents a typical patchy distribution, since it is linked to water bodies inhabited by the lymnaeid snail vectors. In the Northern Bolivian Altiplano, significant differences were detected among the different school surveys as well as among the different community surveys [10], and a direct relationship between fascioliasis infection and the distance between the school and the nearest water bodies representing transmission foci was verified [35].

In the districts analysed in the governorates of Alexandria and Behera, however, freshwater habitats inhabited by lymnaeid snail vectors constituting fascioliasis transmission foci are artificial. The man-made irrigation system is constituted by very numerous canals, including from the main very wide and deep ones directly deriving from the Nile river up to very small ones filled by pumps and reaching each of a myriad of plant cultures distributed everywhere throughout the Nile Delta flatlands. This suggests that familiar household and traditions concerning e.g., dietary habits and individual behaviour regarding infection risk activities [4], should be considered as important factors for infection sources leading to repeated reinfections.

**Figure 7:**
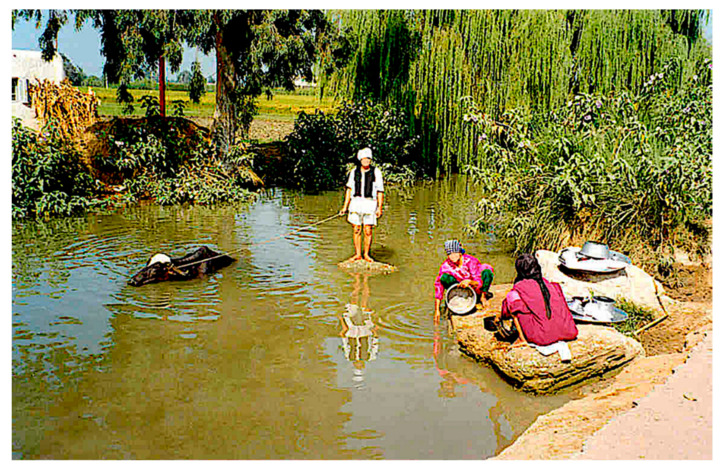
The whole life cycle of the liver fluke in an image from Hosh Esa district: bathing buffalo as animal reservoir; a freshwater collection inhabited by lymnaeid vectors as transmission focus; woman and girl washing kitchen utensils; household (see left background) in the proximity. Photograph by F. Curtale.

An appropriate study of the relationships between human infection by *Fasciola* and the dietary habits and household characteristics of Egyptian inhabitants in the Nile Delta showed that the daily consumption of raw seeds, followed by the presence of piped water in the house, the presence of livestock in the household, the habit to bring the animals to the canal for drinking and/or bathing (Figure 7), as well as the custom of cultivating the vegetables eaten in the household were important risk factors [53]. Entering into water canals to wash foods, dishes, clothes, vegetables and even themselves, as well as drinking water from the smaller irrigation canals bordering the plantations surrounding the outer village suburbs, are additional risky activities. Accumulation of infecting flukes because of the absence of premunition may subsequently give rise to higher infection burdens in given subjects [20,22].

This scenario is consequently different from that in the Bolivian Altiplano hyperendemic area where transmission foci are linked to freshwater collections deriving from water coming from the Eastern Andean Chain [6,33], as well as from the mainly natural transmission foci in the human hyperendemic area in the Peruvian valley of Cajamarca [54,55]. The Egyptian scenario is similar, nevertheless, to the hyperendemic area of the Peruvian Altiplano of Puno linked to a man-made irrigation in Asillo [11], and also to the human infection area linked to the wide irrigation system in the Punjab province of Pakistan [7].

### 3.3. Relationship with Gender

In the previous surveys, carried out in the summer period, no statistically significant differences were detected in intensities between both sexes, although the child presenting the highest intensity in those surveys, 432 epg, was a 9-year-old girl from El Kaza [13]. The absence of intensity differences between males and females was emphasised at that moment, because of the contrast with what is known in Bolivia, where females shed pronouncedly and significantly more eggs than males [10], or in Peru, where the highest overall egg counts were detected in girls [11].

With regard to gender, the analysis of the high intensity cases recorded in the present study shows, even despite the statistical analysis not furnishing a conclusive result, that females are more affected by high burdens than males. A similar although again non-significant result was obtained when analysing 1331 subjects from the Behera governorate [56]. This aspect appears parallel to the significantly higher prevalence in females [13], and also agrees with the result of females shedding significantly more eggs than males obtained when analysing low and moderate intensities in a very large sample of 21,477 subjects [44]. Thus, both prevalence and intensity pictures in the Nile Delta lowlands in Egypt follow the same gender-related patterns described in Andean highlands of Bolivia and Peru [10,11].

### 3.4. Relationship with Age

Concerning age, egg counts higher than 400 epg seem to concentrate on children, given that no adult subjects have ever been detected shedding such high epg in surveys including total population surveys performed in Egypt so far. This is similar to the Northern Bolivian Altiplano, where data obtained in subjects older than 20 years showed that epg counts decrease as the age increases [10], and also to the Peruvian area of Puno where the highest overall egg counts were detected in the youngest age group [11].

The age curve of the high burden cases studied shows a peak similar to what is known in Bolivia and Peru. However, in the Nile Delta region this high intensity peak appears in the 7–10 year-old group with most cases in the 9-year old group. This appears to be somewhat before the peak observed in Andean countries, where it is at the 9–11 year-old group [10,11].

When analysing the individual cases, the cause for such an earlier peak in Egypt when compared to Bolivia and Peru appears related to a gender factor, as it becomes evident that the majority of younger (6–9-year old) children harbouring high burdens are females. This precocity of girls presenting this peak a little bit earlier than boys is worth mentioning. Such a difference in this age peak between males and females found in Egypt has never been reported elsewhere. The reason for such gender difference remains unknown, but it has tentatively been linked to differences in behaviour and traditions between Egyptian boys and girls [44,53,56]. This also contrasts with the higher infection in males than in females in short aged, preschool children recently highlighted in a worldwide analysis of liver fluke infection in such small children [57].

### 3.5. Observations on Seasonality

Contrary to Andean altiplanos, where fascioliasis transmission occurs all year long [33,35,58], the transmission of this disease follows a bi-seasonal pattern throughout the circum-Mediterranean region [59]. It is also seasonal in Peruvian valleys [60]. Experimental studies on Mediterranean liver flukes showed that the level of epg shedding peaks in spring and autumn. This chronobiological pattern appears to favour parasite transmission because of the seasonality of the Mediterranean lymnaeid populations [46].

In Egypt, prevalences of fascioliasis in the Nile Delta region have also been verified to follow seasonality, both in animals [45] and humans [61]. The maximum snail infection rate was observed in June and July, while the number of acute human infections was reported to peak in August [61]. Summer has been noted to be the highest infection season in which both animals and humans present the highest prevalences [48].

However, the results of the present study show that the number of high intensity cases in winter (February and March) are remarkably higher than those found in summer (June) using the same methodology and quantitative diagnostic technique in several of the districts here analysed [13]. According to the liver fluke life cycle chronology , most of those children presenting high burdens should have been infected in the previous autumn period of transmission. This disagreement between prevalences peaking in summer whereas intensities peaking in winter may be due to reinfections. Many of these high burden cases may be the consequence of repeated and accumulative reinfections. Indeed, there is no premunition in fascioliasis [21], reinfections are known to be frequent in human fascioliasis hyperendemic areas [20], and increases in egg shedding have recently been experimentally proven in reinfections occurring during the chronic phase of the disease [22].

### 3.6. Observations on Schistosomiasis Coinfection

Finally, the fascioliasis-schistosomiasis coinfection deserves to be emphasised given the pathogenicity implications of this coinfection in children. The coinfection with *S. mansoni* in 25% of the children presenting high fascioliasis intensities further supports the significant positive association previously detected in Egypt between these two trematode diseases in total population surveys [13,44]. Indeed, planorbid snails such as *Bulinus truncatus* transmitting the aforementioned schistosome species are frequently found coexisting with lymnaeid vector species in the same freshwater collections throughout the Nile Delta region.

The present paper, moreover, highlights the high morbidity impact of this association in children already suffering from marked fasciolid pathogenicity due to the presence of high liver fluke burdens.

### 3.7. Repercussions on Health

A higher number of infecting flukes is known to be more pathogenic for the patient. The degree of pathological effects in human fascioliasis depends primarily on the number of flukes [8], which increases the severity of infection in the chronic and advanced chronic phases of the disease [62,63,64]. Consequently, high morbidity due to such heavy infections might be expected in this vulnerable group.

Total fluke volume in a patient is another factor proved to be linked to pathogenicity [62,63,64], i.e., bigger flukes are more harmful. In the Nile Delta region, *F. hepatica*, *F. gigantica* and intermediate hybrid forms between the two “pure” phenotypes are present [52]. Fluke specimens of both *F. gigantica* and intermediate hybrid forms are bigger than those of *F. hepatica*. Consequently, Egyptian children are exposed to a higher pathogenicity risk than children living in endemic areas where only *F. hepatica* is present, such as in Bolivia and Peru.

Liver fluke pathogenicity has also been experimentally proven to be related to the length of the chronic period of the disease [62,63], i.e., long-term infections are more harmful. In rural areas, children are usually not guided by parents to health centres for diagnosis during the acute phase, and the chronic phase may develop asymptomatically or with only moderate symptoms and thus remain undiagnosed and untreated. This highlights the importance of the active detection of infection cases, including diagnosis and treatment, as already ongoing in the Nile Delta governorates. The strategy of selective treatment implemented in Egypt [43] reduces the chances to develop drug resistance and, as a collateral benefit, brings the attention to the diagnosis of other parasites in the screened children, which is not the case in the mass treatment distribution strategy.

There is a risk of colic during the treatment of such cases harbouring a high number of flukes. Treatment with the normal dose may induce a potential bile duct obstruction by dead flukes swept along. Therefore, the determination of the intensity in heavy infections in the patient’s diagnosis becomes crucial to establish the doses for a treatment course appropriate to avoid a colic [26]. Children with epg counts higher than 400 should be treated with 12-h-spaced reduced triclabendazole doses and additionally hospitalised for post-treatment follow-up to prevent potential colic episodes. In the Nile Delta governorates, special attention should be therefore given to subjects presenting several eggs in the Kato-Katz slides. Exact epg should be calculated and the aforementioned security measures should be taken in the cases of more than 400 epg. Moreover, it should be here noted that neither serological tests nor coproantigen tests allow for burden assessments [28,65].

The immunosuppression effect in the chronic and advanced chronic phases of fascioliasis [19,21] is in the background of co-infections with other parasitic and infectious diseases and subsequent higher morbidity in co-infected patients [15]. Assessing coinfections in these heavy infected children is therefore crucial and the use of at least an additional diagnostic technique for the detection of other protozoans and helminths is recommended in these cases [65]. Moreover, it should be considered that coinfections with given parasites may interfere in the treatment efficacy and lead to the need of an additional dose [66].

The new data of the present study on high burdens in children should be taken into account when estimating both short and long-term fascioliasis impact by calculating the Disability Adjusted Life Years (DALYs) [67] for the very wide Nile Delta region in Egypt.

## 4. Concluding Remarks

In developed countries, patients are diagnosed in hospitals or other health centres usually during the acute phase or at the beginning of the chronic phase [65]. On the contrary, infected subjects detected in surveys in human endemic areas of developing countries such as Egypt, Bolivia and Peru, are mainly in the advanced stage of chronicity, when not already re-infected due to the high contamination risk [65,68]. As the liver fluke is able to survive many years in human beings and liver fluke infection may occur very early in life, even in small children aged less than 1 year [57], pronounced high burdens as those found in Egypt in children of less than 14 years old mostly suggest accumulative reinfections rather than the ingestion of numerous metacercariae at once. 

The impact of this epidemiological scenario in the Nile Delta on children with high liver fluke burdens is of a great public health importance owing to:

- Liver fluke infection intensities found in the 96 schoolchildren from the governorates of Alexandria and Behera, higher than 400 epg and up to 2304 epg, are the highest intensities hitherto reported not only in Egypt but also in the whole Old World.
- The lower frequency of such high intensity cases in Egypt when compared to those in Bolivia and Peru suggests the difference in the Nile Delta lowlands to be in part related to the high-altitude-enhanced fasciolid transmission observed in the Andean highlands.
- The high intensities here reported in children highlight a problem of very high infection burdens concentrated in infancy, with their consequent higher pathogenicity, morbidity and underdevelopment of individuals and communities.
- In subjects in whom numerous liver fluke eggs are observed in a qualitative diagnostic analysis, exact epg counts should be assessed and preventive measures should be taken in the cases of more than 400 epg, by treating with reduced triclabendazole spaced doses and by additional hospitalisation for post-treatment follow-up enabling quick reaction if potential colic episodes appear.

## 5. Materials and Methods

### 5.1. Study Population

Human cases analysed were detected in coprological surveys of schoolchildren from districts of the governorates of Alexandria and Behera (Figure 1), within activities performed as part of a school health program related to integrated helminth control at governorate level in Egypt [69]. In these districts, fascioliasis had previously proved to be an important public health problem because of the relatively high number of infected children diagnosed [13,31,70]. The level of district has been used in the present study to obtain groupings including a sufficiently high number of children presenting high intensities as to allow for significant analyses of such cases.

Kato-Katz slides performed in the preliminary diagnostic analyses by the Alexandria and Behera survey teams of the Egyptian MoHP were re-examined for exact egg counting, especially focusing on those slides including several *Fasciola* eggs per slide (e.g., slides noted as “more than 10 eggs”, “++” or “+++”). These slides concerned stool samples of a total of 6657 children, including 362 positive (5.4%) from 42 schools and villages in the following governorates and districts:

Alexandria governorate:

- 123 children from Wasat-Abis 8 district: (1) Abdel Aziz Haroun, Abis 8; (2) Suzanne Mubarak, Abis 8;
- 164 children from Waqad-Haris district: (3) Ola Garbea, Ola Gharbea; (4) El Missiry, El Missiry.

Behera governorate:

- 815 children from Abu El Matamir district: (5) Abu El Matamir, Abu El Bahany; (6) Bahany, Abu El Bahany; (7) Amen Aloba, Abu El Bahany; (8) El Ezba El Hamraa, Abu El Bahany; (9) Abd El Malek El Sayed, Abu El Bahany; (10) Barakat Abd El Malek, Abu El Bahany;
- 836 children from Abu Homos district: (11) Besentway, Besentway; (12) Demesna, Demesna;
- 250 children from Damanhour district: (13) Dorbok, Dorbok;
- 886 children from Delengat district: (14) Yunis Hemeda, Delengat; (15) Azhar Delengat, Delengat; (16) Abu Baki, Delengat; (17) Wagaa Sad, Delengat; (18) Sad Lohaeem, Delengat; (19) El Saieda Fatma, Delengat; (20) Azhar, Tiba; (21) Al Khelalia, Al Khelalia;
- 1284 children from Hosh Esa district: (22) Feraz, Feraz; (23) El Dermerdash, El Kaza; (24) El Roda, El Kaza; (25) Mehress, El Kaza; (26) El Sotomia, El Kaza; (27) El Bostan, Kobry-Abd; (28) Harara, Harara; (29) Azhar Harara, Harara; (30) El Hadad El Bahany, El Hadad; (31) El Hadad El Westany, El Hadad; (32) Abu Malout, El Karnin; (33) El Kardood, El Karnin; (34) Richo, Richo;
- 1882 children from Kafr El Dawar district: (35) Abis 1, Abis 1; (36) Abis 5, Abis 5; (37) El Shahed Saied Sarhan, Kom El Berka; (38) Azhar Bolin, Bolin; (39) Kombaniat Loken, Zuhra; (40) El Malka, Zuhra;
- 417 children from Rahmanea district: (41) Somekrat, Somekrat; (42) Azhar, Menet Salama.

The geographical location of these governorates and districts is shown in Figure 1 and the distribution of the children whose Kato-Katz slides could be re-checked are detailed in Table 1. It should be emphasised that the positivity percentages included in Table 1 should not be considered as conclusive prevalence data, but only as information about the subjects whose egg outputs could be re-evaluated. The relevant information furnished by these infection positivity results was, moreover, already analysed in detail in a previous study [44].

### 5.2. Stool Collection and Laboratory Methods

A clean, plastic, wide-mouth, numbered container with a snap-on lid was given to every child. All children were then asked to try to fill the container with their own faeces and to return it. One stool sample per subject was collected and personal data (name, sex, and age) were noted on delivery of the container. Faecal specimens were transported to the laboratories of the Regional Health Directorate of the MoHP in Alexandria and the Behera Regional Health Office in Damanhour within 1–3 h of collection.

In these laboratories, the infection of each subject was assessed through the preparation of three Kato-Katz slides (Helm-Test, AK test, AK Industria e Comercio Ltda, Belo Horizonte, Brazil) made from each stool sample following WHO recommendations, using a standard template delivering about 41.7 mg of faeces [29,30]. These slides were initially examined within 1 h of preparation. Positive cases were treated with triclabendazole for human use (Egaten^®^) [43], a drug registered in Egypt [71] and which is the drug of election for human fascioliasis at present [72].

Taking into account the problem posed in specific egg measurements by the coexistence of *F. hepatica*, *F. gigantica* and intermediate forms [73] in the whole human fascioliasis endemic area throughout the Nile Delta [52], no attempt to differentiate fasciolid species by egg measurements was made.

Positive Kato-Katz slides presenting several eggs were later re-examined for exact egg counting to assess individual intensities in the cases of high egg numbers. The number of eggs per slide were converted to epg using a multiplication factor of 24. Exact quantification was focused on stool samples of children presenting more than 400 epg. This cut-off of 400 epg was taken to differentiate between high intensity (high ≥ 400 epg) and other intensities (low or moderate), according to the guidelines of the World Health Organisation [26] which follow the intensity classification for schistosomiasis mansoni [34]. The importance of this cut-off relies on the patient burden to be considered when establishing the adequate treatment procedure. Patients with an epg count higher than 400 are in need to be hospitalised for prevention follow-up of potential post-treatment colics caused by biliary obstruction due to drag and subsequent accumulation of a high number of liver fluke specimens [27,28]. In such cases, the recommended treatment should include 12 h-spacing of reduced doses.

Because of the pathogenicity importance of the coinfections of fascioliasis with schistosomiasis in children living in governorates of the Nile Delta [14], the additional detection of eggs of *Schistosoma* species in the re-examined Kato-Katz slides was duly noted.

### 5.3. Data Management and Statistical Analysis

Intensity of infection was calculated and presented as ranges as well as in arithmetic and geometric means. Geometric means were calculated after individual egg counts had been converted according to the log (n + 1) transformation [74]. Statistical analyses were performed using SPSS Statistics 26 software (IBM, Armonk, NY, USA). To detect differences in intensity according to gender, the t-test was performed using the converted epg data. For the detection of differences in intensity according to age and to districts, the Kruskal–Wallis test was carried out using the normal epg data. Analyses for differences according to districts were conducted only for Behera, since the sample size in Alexandria was too small.

Statistical comparison of categorical variables was carried out with the Fisher’s exact test, Chi-square test and Yates continuity corrected Chi-square test.

### 5.4. Study Limitations

Whereas the sample size of heavy infected children (n = 96) is sufficient to demonstrate that the fascioliasis transmission in the western Nile Delta gives rise to individual cases of intensities higher than 400 epg in which care should be taken in the treatment to avoid a colic, as well as to analyse their relationships with gender and age, there are limitations regarding geographical distribution and seasonality. The number of high intensity cases is too small as to enable a statistical analysis according to the numerous localities whose schools were surveyed. Additionally, schools are not comparable regarding the size of the surrounding area of the attending children. Due to overdispersion caused by the homogeneous transmission scenarios underlying a similar infection risk throughout, the aforementioned number of cases only proved to allow for statistically significant analyses when considering the geographical level of district. Concerning seasonality, although interesting observations may be noted, the transversal sample collection was organised to detect infected children for their subsequent treatments, according to the health priorities and objectives of the international financing projects. Therefore, surveys could unfortunately not follow a longitudinal continuous monthly sampling in given localities allowing for the appropriate analysis of seasonal influences.

## Figures and Tables

**Table 1:** Distribution of the 96 fascioliasis-infected children presenting intensities higher than 400 epg, according to governorates and districts surveyed in the Nile Delta region, Egypt.

Districts	No. of Intensity Re-Checked Children	Children with Several Eggs per Slide *		No. Children with epg >400 (%)	epg		
		No.	%		Range	Arithm. Mean	Geom. Mean
**Governorate of Alexandria**							
Wasat-Abis 8	123	6	4.9	3 (2.4%)	408–2040	1072.0	861.4
Waqad-Haris	164	9	5.4	3 (1.8%)	408–432	416.0	415.9
**Governorate of Behera**							
Abu El Matamir	815	52	6.4	9 (1.1%)	408–1008	650.7	618.2
Abu Homos	836	56	6.6	10 (1.2%)	456–1680	832.8	751.9
Damanhour	250	8	0.3	1 (0.4%)	576	576.0	576.0
Delengat	886	58	6.5	9 (1.0%)	408–1752	621.3	547.8
Hosh Esa	1284	95	7.4	34 (2.6%)	408–2304	730.6	650.5
Kafr El Dawar	1882	64	3.4	25 (1.3%)	408–1752	659.5	612.3
El Rahmannia	417	15	3.6	2 (0.5%)	456–552	504.0	501.7
TOTAL	6657	362	5.4	96 (1.4%)	408–2304	673.6	615.1

epg = eggs per gram of faeces. * = slides noted as “more than 10 eggs”, “++” or “+++” in previous qualitative diagnostic analysis.

**Table 2:** Distribution of high fascioliasis intensities (more than 400 eggs per gram) detected in stool samples of schoolchildren from Alexandria and Behera governorates, Egypt, according to sex and age.

epg	Cases No. (%)	Gender		Age (Years)							
		Males	Females	6 y	7 y	8 y	9 y	10 y	11 y	12 y	13 y
		No. (%)	No. (%)	No. (%)	No. (%)	No. (%)	No. (%)	No. (%)	No. (%)	No. (%)	No. (%)
408	12 (12.5)	4 (10.5)	8 (13.8)	2 (14.3)	2 (10.5)	2 (10.0)	1 (5.3)	2 (11.8)	1 (25.0)	2 (100.0)	––
432	11 (11.5)	5 (13.2)	6 (10.3)	––	3 (15.8)	––	4 (21.1)	3 (17.6)	1 (25.0)	––	––
456	9 (9.4)	2 (5.3)	7 (12.1)	––	1 (5.3)	2 (10.0)	4 (21.1)	2 (11.8)	––	––	––
480	7 (7.3)	4 (10.5)	3 (5.2)	––	3 (15.8)	1 (5.0)	2 (10.5)	1 (5.9)	––	––	––
504	4 (4.2)	1 (2.6)	3 (5.2)	2 (14.3)	1 (5.3)	––	1 (5.3)	––	––	––	––
528	3 (3.1)	2 (5.3)	1 (1.7)	1 (7.14)	––	1 (5.0)	––	1 (5.9)	––	––	––
552	4 (4.2)	1 (2.6)	3 (5.2)	––	2 (10.5)	1 (5.0)	––	1 (5.9)	––	––	––
576	4 (4.2)	1 (2.6)	2 (3.4)	––	1 (5.3)	––	1 (5.3)	2 (11.8)	––	––	––
600	2 (2.1)	1 (2.6)	1 (1.7)	––	––	1 (5.0)	––	1 (5.9)	––	––	––
624	1 (1.0)	1 (2.6)	0 (0.0)	––	––	1 (5.0)	––	––	––	––	––
648	4 (4.2)	2 (5.3)	2 (3.4)	2 (14.3)	––	1 (5.0)	1 (5.3)	––	––	––	––
672	3 (3.1)	1 (2.6)	2 (3.4)	––	1 (5.3)	1 (5.0)	––	1 (5.9)	––	––	––
696	1 (1.0)	1 (2.6)	0 (0.0)	––	––	––	––	––	1 (25.0)	––	––
720	4 (4.2)	1 (2.6)	3 (5.2)	1 (7.14)	1 (5.3)	2 (10.0)	––	––	––	––	––
744	1 (1.0)	0 (0.0)	1 (1.7)	––	––	––	1 (5.3)	––	––	––	––
768	3 (3.1)	2 (5.3)	1 (1.7)	1 (7.14)	––	––	––	––	1 (25.0)	––	1 (100)
792	2 (2.1)	2 (5.3)	0 (0.0)	1 (7.14)	––	1 (5.0)	––	––	––	––	––
816	1 (1.0)	0 (0.0)	1 (1.7)	––	1 (5.3)	––	––	––	––	––	––
864	1 (1.0)	0 (0.0)	1 (1.7)	1 (7.14)	––	––	––	––	––	––	––
888	1 (1.0)	0 (0.0)	1 (1.7)	––	1 (5.3)	––	––	––	––	––	––
936	2 (2.1)	2 (5.3)	0 (0.0)	––	1 (5.3)	1 (5.0)	––	––	––	––	––
984	1 (1.0)	0 (0.0)	1 (1.7)	––	––	––	––	1 (5.9)	––	––	––
1008	1 (1.0)	0 (0.0)	1 (1.7)	––	1 (5.3)	––	––	––	––	––	––
1056	1 (1.0)	0 (0.0)	1 (1.7)	1 (7.14)	––	––	––	––	––	––	––
1080	1 (1.0)	0 (0.0)	1 (1.7)	––	––	––	1 (5.3)	––	––	––	––
1152	1 (1.0)	0 (0.0)	1 (1.7)	––	––	1 (5.0)	––	––	––	––	––
1224	1 (1.0)	0 (0.0)	1 (1.7)	1 (7.14)	––	––	––	––	––	––	––
1296	2 (2.1)	0 (0.0)	2 (3.4)	––	––	1 (5.0)	––	1 (5.9)	––	––	––
1320	1 (1.0)	1 (2.6)	0 (0.0)	1 (7.14)	––	––	––	––	––	––	––
1344	1 (1.0)	0 (0.0)	1 (1.7)	––	––	1 (5.0)	––	––	––	––	––
1560	1 (1.0)	1 (2.6)			––	1 (5.0)	––	––	––	––	––
1680	1 (1.0)	0 (0.0)	1 (1.7)	––	––	––	1 (5.3)	––	––	––	––
1752	2 (2.1)	1 (2.6)	1 (1.7)	––	––	––	1 (5.3)	1 (5.9)	––	––	––
2040	1 (1.0)	1 (2.6)	0 (0.0)	––	––	1 (5.0)	––	––	––	––	––
2304	1 (1.0)	0 (0.0)	1 (1.7)	––	––	––	1 (5.3)	––	––	––	––
Total	96 (100)	38 (39.6)	58 (60.4)	14 (14.6)	19 (19.8)	20 (20.8)	19 (19.8)	17 (17.7)	4 (4.2)	2 (2.1)	1 (1.0)

epg = eggs per gram of faeces. –– means that no child of this age presented this number of eggs.

**Table 3:** Number of fascioliasis-infected children presenting high epg counts according to intensity groups.

Epg Counts	Children			Males		Females	
Groups	No.	%	Mean	No.	%	No.	%
400–699	65	67.7	8.5	27	41.5	38	58.5
700–999	16	16.7	7.9	7	43.8	9	56.3
1000–1299	7	7.3	7.7	0	0	7	100.0
1300–1599	4	4.2	7.8	2	50.0	2	50.0
1600–1899	2	2.1	9.5	1	50.0	1	50.0
≥1900	2	2.1	8.5	1	50.0	1	50.0

epg = eggs per gram of faeces; % of children = calculated in relation to the total number of 96 presenting high intensities; mean = mean age in each intensity group; % of males and females = calculated for each intensity group.

## Data Availability

Datasets generated for this study are available on request to the corresponding authors.

## References

[1] Mas-Coma S., Valero M.A., Bargues M.D. (2009). *Fasciola*, lymnaeids and human fascioliasis, with a global overview on disease transmission, epidemiology, evolutionary genetics, molecular epidemiology and control. Adv. Parasitol..

[2] Bargues M.D., Artigas P., Mera y Sierra R., Pointier J.P., Mas-Coma S. (2007). Characterisation of *Lymnaea cubensis*, *L. viatrix* and *L. neotropica* n. sp., the main vectors of *Fasciola hepatica* in Latin America, by analysis of their ribosomal and mitochondrial DNA. Ann. Trop. Med. Parasitol..

[3] Bargues M.D., Artigas P., Khoubbane M., Flores R., Glöer P., Rojas-Garcia R., Ashrafi K., Falkner G., Mas-Coma S. (2011). *Lymnaea schirazensis*, an overlooked snail distorting fascioliasis data: Genotype, phenotype, ecology, worldwide spread, susceptibility, applicability. PLoS ONE.

[4] Mas-Coma S., Bargues M.D., Valero M.A. (2018). Human fascioliasis infection sources, their diversity, incidence factors, analytical methods and prevention measures. Parasitology.

[5] Afshan K., Fortes-Lima C.A., Artigas P., Valero M.A., Qayyum M., Mas-Coma S. (2014). Impact of climate change and man-made irrigation systems on the transmission risk, long-term trend and seasonality of human and animal fascioliasis in Pakistan. Geospat. Health.

[6] Bargues M.D., Artigas P., Angles R., Osca D., Duran P., Buchon P., Gonzales-Pomar R.K., Pinto-Mendieta J., Mas-Coma S. (2020). Genetic uniformity, geographical spread and anthropogenic habitat modifications of lymnaeid vectors found in a One Health initiative in the highest human fascioliasis hyperendemic of the Bolivian Altiplano. Parasit. Vectors.

[7] Mas-Coma S. (2020). Human fascioliasis emergence risks in developed countries: From individual patients and small epidemics to climate and global change impacts. Enferm. Infecc. Microbiol. Clin..

[8] Chen M.G., Mott K.E. (1990). Progress in assessment of morbidity due to *Fasciola hepatica* infection: A review of recent literature. Trop. Dis. Bull..

[9] Hillyer G.V., Soler de Galanes M., Rodriguez-Perez J., Bjorland J., Silva de Lagrava M., Ramirez Guzman S., Bryan R.T. (1992). Use of the Falcon Assay Screening Test—Enzyme-Linked Immunosorbent Assay (FAST-ELISA) and the Enzyme-Linked Immunoelectrotransfer Blot (EITB) to determine the prevalence of human Fascioliasis in the Bolivian Altiplano. Am. J. Trop. Med. Hyg..

[10] Esteban J.G., Flores A., Angles R., Mas-Coma S. (1999). High endemicity of human fascioliasis between Lake Titicaca and La Paz valley, Bolivia. Trans. R. Soc. Trop. Med. Hyg..

[11] Esteban J.G., Gonzalez C., Bargues M.D., Angles R., Sanchez C., Naquira C., Mas-Coma S. (2002). High fascioliasis infection in children linked to a man-made irrigation zone in Peru. Trop. Med. Int. Health.

[12] Gonzalez L.C., Esteban J.G., Bargues M.D., Valero M.A., Ortiz P., Naquira C., Mas-Coma S. (2011). Hyperendemic human fascioliasis in Andean valleys: An altitudinal transect analysis in children of Cajamarca province, Peru. Acta Trop..

[13] Esteban J.G., Gonzalez C., Curtale F., Muñoz-Antoli C., Valero M.A., Bargues M.D., El Sayed M., El Wakeel A., Abdel-Wahab Y., Montresor A. (2003). Hyperendemic fascioliasis associated with schistosomiasis in villages in the Nile Delta of Egypt. Am. J. Trop. Med. Hyg..

[14] Mera y Sierra R., Agramunt V.H., Cuervo P., Mas-Coma S. (2011). Human fascioliasis in Argentina: Retrospective overview, critical analysis and baseline for future research. Parasit. Vectors.

[15] Mas-Coma S., Agramunt V.H., Valero M.A. (2014). Neurological and ocular fascioliasis in humans. Adv. Parasitol..

[16] Gonzalez-Miguel J., Valero M.A., Reguera-Gomez M., Mas-Bargues C., Bargues M.D., Simon-Martin F., Mas-Coma S. (2019). Numerous *Fasciola* plasminogen-binding proteins may underlie blood-brain barrier leakage and explain neurological disorder complexity and heterogeneity in the acute and chronic phases of human fascioliasis. Parasitology.

[17] Rondelaud D., Dreyfuss G., Vignoles P. (2006). Clinical and biological abnormalities in patients after fasciolosis treatment. Med. Mal. Infect..

[18] Dalton J.P., Robinson M.W., Mulcahy G., O’Neill S.M., Donnelly S. (2013). Immunomodulatory molecules of *Fasciola hepatica*: Candidates for both vaccine and immunotherapeutic development. Vet. Parasitol..

[19] Aldridge A., O’Neill S.M. (2016). *Fasciola hepatica* tegumental antigens induce anergic like T cells via dendritic cells in a mannose receptor dependent manner. Eur. J. Immunol..

[20] Valero M.A., Perez-Crespo I., Chillon-Marinas C., Khoubbane M., Quesada C., Reguera-Gomez M., Mas-Coma S., Fresno M., Girones N. (2017). *Fasciola hepatica* reinfection potentiates a mixed Th1/Th2/Th17/Treg response and correlates with the clinical phenotypes of anemia. PLoS ONE.

[21] Girones N., Valero M.A., Garcia-Bodelon M.A., Chico-Calero M.I., Punzon C., Fresno M., Mas-Coma S. (2007). Immune suppression in advanced chronic fascioliasis: An experimental study in a rat model. J. Infect. Dis..

[22] Valero M.A., Girones N., Reguera-Gomez M., Perez-Crespo I., Lopez-Garcia M.P., Quesada C., Bargues M.D., Fresno M., Mas-Coma S. (2020). Impact of fascioliasis reinfection on *Fasciola hepatica* egg shedding: Relationship with the immune-regulatory response. Acta Trop..

[23] World Health Organization (2013). Sustaining the Drive to Overcome the Global Impact of Neglected Tropical Diseases.

[24] World Health Organization (2020). Ending the Neglect to Attain the Sustainable Development Goals. A Road Map for Neglected Tropical Diseases 2021–2030.

[25] Curtale F. (2008). Treatment of human fascioliasis with triclabendazole: Good news. Trans. R. Soc. Trop. Med. Hyg..

[26] World Health Organization (2007). Report of the WHO Informal Meeting on Use of Triclabendazole in Fascioliasis Control.

[27] Villegas F., Angles R., Barrientos R., Barrios G., Valero M.A., Hamed K., Grueningr H., Ault S.K., Montresor A., Engels D. (2012). Administration of triclabendazole is safe and effective in controlling fascioliasis in an endemic community of the Bolivian Altiplano. PLoS Negl. Trop. Dis..

[28] Valero M.A., Periago M.V., Perez-Crespo I., Angles R., Villegas F., Aguirre C., Strauss W., Espinoza J.R., Herrera P., Terashima A. (2012). Field evaluation of a coproantigen detection test for fascioliasis diagnosis and surveillance in human hyperendemic areas of Andean countries. PLoS Negl. Trop. Dis..

[29] Katz N., Chaves A., Pellegrino J. (1972). A simple device for quantitative stool thick-smear technique in *Schistosomiasis mansoni*. Rev. Inst. Med. Trop. Sao Pãulo.

[30] Ash L.R., Orihel T.C., Savioli L. (1994). Bench Aids for the Diagnosis of Intestinal Parasites.

[31] Curtale F., Nabil M., El Wakeel A., Shamy M.Y., Behera Survey Team (1998). Anaemia and intestinal parasitic infections among school age children in Behera Governorate, Egypt. J. Trop. Ped..

[32] Mas-Coma S., Funatsu I.R., Angles R., Buchon P., Mas-Bargues C., Artigas P., Valero M.A., Bargues M.D. (2021). Domestic pig prioritized in one health action against fascioliasis in human endemic areas: Experimental assessment of transmission capacity and epidemiological evaluation of reservoir role. One Health.

[33] Bargues M.D., Angles R., Coello J., Artigas P., Funatsu I.R., Cuervo P.F., Buchon P., Mas-Coma S. (2021). One Health initiative in the Bolivian Altiplano human fascioliasis hyperendemic area: Lymnaeid biology, population dynamics, microecology and climatic factor influences. Braz. J. Vet. Parasitol..

[34] Montresor A., Crompton D.W.T., Bundy D.A.P., Hall A., Savioli L. (1998). Guidelines for the Evaluation of Soil-Transmitted Helminthiasis and Schistosomiasis at Community Level. A Guide for Managers of Control Programmes.

[35] Mas-Coma S., Angles R., Esteban J.G., Bargues M.D., Buchon P., Franken M., Strauss W. (1999). The Northern Bolivian Altiplano: A region highly endemic for human fascioliasis. Trop. Med. Int. Health.

[36] O’Neill S.M., Parkinson M., Strauss W., Angles R., Dalton J.P. (1998). Immunodiagnosis of *Fasciola hepatica* (fascioliasis) in a human population in the Bolivian Altiplano using purified cathepsin L cysteine proteinase. Am. J. Trop. Med. Hyg..

[37] Mas-Coma S., Funatsu I.R., Bargues M.D. (2001). *Fasciola hepatica* and lymnaeid snails occurring at very high altitude in South America. Parasitology.

[38] Valero M.A., Perez-Crespo I., Khoubbane M., Artigas P., Panova M., Ortiz P., Maco V., Espinoza J.R., Mas-Coma S. (2012). *Fasciola hepatica* phenotypic characterisation in Andean human endemic areas: Valley versus altiplanic patterns analysed in liver flukes from sheep from Cajamarca and Mantaro, Peru. Infect. Genet. Evol..

[39] Curtale F., Hammoud E.S., El Wakeel A., Mas-Coma S., Savioli L. (2000). Human fascioliasis, an emerging public health problem in the Nile Delta, Egypt. Res. Rev. Parasitol..

[40] Haseeb A.N., El Shazly A.M., Arafa M.A.S., Morsy A.T.A. (2002). A review on fascioliasis in Egypt. J. Egypt. Soc. Parasitol.

[41] World Health Organization (1995). Control of Foodborne Trematode Infections.

[42] El Shazly A.M., El-Nahas H.A., Abdel-Mageed A.A., El Beshbishi S.N., Azab M.S., Abou El Hasan M., Arafa W.A.S., Morsy T.A. (2005). Human fascioliasis and anaemia in Dakhalia governorate, Egypt. J. Egypt. Soc. Parasitol..

[43] Curtale F., Hassanein Y.A.E., Savioli L. (2005). Control of human fascioliasis by selective chemotherapy: Design, cost and effect of the first public health, school-based intervention implemented in endemic areas of the Nile Delta, Egypt. Trans. R. Soc. Trop. Med. Hyg..

[44] Curtale F., Hassanein Y.A., Barduagni P., Yousef M.M., Wakeel A.E., Hallaj Z., Mas-Coma S. (2007). Human fascioliasis infection: Gender differences within school-age children from endemic areas of the Nile Delta, Egypt. Trans. R. Soc. Trop. Med. Hyg..

[45] Soliman M.S., Angelico M., Rocchi G.G. (1998). Control of veterinary fascioliasis. Infectious Diseases and Public Health. A Research and Clinical Update.

[46] Valero M.A., Panova M., Comes A.M., Fons R., Mas-Coma S. (2002). Patterns in size and shedding of *Fasciola hepatica* eggs by naturally and experimentally infected murid rodents. J. Parasitol..

[47] Esteban J.G., Flores A., Aguirre C., Strauss W., Angles R., Mas-Coma S. (1997). Presence of very high prevalence and intensity of infection with *Fasciola hepatica* among Aymara children from the Northern Bolivian Altiplano. Acta Trop..

[48] El Bahy M.M. (1997). Fascioliasis among animal, snail and human hosts in Kafr El-Sheikh Governorate with special reference to species infecting humans. Vet. Med. J. Giza.

[49] Massoud A.M.A., El-Kholy N.M.B., El-Shennawy F.A., Farag R.E. (2004). Study of some immune aspects in patients with fascioliasis before and after *Chommiphora molmol* (Mirazid) treatment. J. Egypt. Soc. Parasitol..

[50] El Shazly A.M., Soliman M., Gabr A., Haseeb A.N., Morsy A.T.A., Arafa M.A.S., Morsy T.A. (2001). Clinico-epidemiological study of human fascioliasis in an endemic focus in Dakahlia governorate, Egypt. J. Egypt. Soc. Parasitol..

[51] Esteban J.G., Flores A., Angles R., Strauss W., Aguirre C., Mas-Coma S. (1997). A population-based coprological study of human fascioliasis in a hyperendemic area of the Bolivian Altiplano. Trop. Med. Int. Health.

[52] Periago M.V., Valero M.A., El Sayed M., Ashrafi K., El Wakeel A., Mohamed M.Y., Desquesnes M., Curtale F., Mas-Coma S. (2008). First phenotypic description of *Fasciola hepatica*/*Fasciola gigantica* intermediate forms from the human endemic area of the Nile Delta, Egypt. Infect. Genet. Evol..

[53] Curtale F., Mas-Coma S., Hassanein Y.A.E.W., Barduagni P., Pezzotti P., Savioli L. (2003). Clinical signs and household characteristics associated with human fascioliasis among rural population in Egypt: A case-control study. Parassitologia.

[54] Bargues M.D., Artigas P., Khoubbane M., Ortiz P., Naquira C., Mas-Coma S. (2012). Molecular characterisation of *Galba truncatula*, *Lymnaea neotropica* and *L. schirazensis* from Cajamarca, Peru and their potential role in transmission of human and animal fascioliasis. Parasit. Vectors.

[55] Bardales-Valdivia J.N., Bargues M.D., Hoban-Vergara C., Bardales-Bardales C., Goicoechea-Portal C., Bazan-Zurita H., Del Valle-Mendoza J., Ortiz P., Mas-Coma S. (2021). Spread of the fascioliasis endemic area assessed by seasonal follow-up of rRNA ITS-2 sequenced lymnaeid populations in Cajamarca, Peru. One Health.

[56] Curtale F., Hassanein Y.A.E., El Wakeel A., Mas-Coma S., Montresor A. (2003). Distribution of human fascioliasis by age and gender among rural population in the Nile Delta, Egypt. J. Trop. Ped..

[57] De N.V., Le T.H., Agramunt V.H., Mas-Coma S. (2020). Early postnatal and preschool age infection by *Fasciola* spp.: Report of five cases from Vietnam and worldwide review. Am. J. Trop. Med. Hyg..

[58] Fuentes M.V., Malone J.B., Mas-Coma S. (2001). Validation of a mapping and predicting model for human fasciolosis transmission in Andean very high altitude endemic areas using remote sensing data. Acta Trop..

[59] Ollerenshaw C.B. (1973). A comment on the epidemiology of *Fasciola hepatica* in Italy. Ann. Fac. Med. Vet..

[60] Claxton J.R., Sutherst J., Ortiz P., Clarkson M.J. (1999). The effect of cyclic temperatures on the growth of *Fasciola hepatica* and *Lymnaea viatrix*. Vet. J..

[61] Farag H.F., Salem A., Khalil S.S., Farahat A. (1993). Studies on human fascioliasis in Egypt. 1—Seasonality of transmission. J. Egypt. Soc. Parasitol..

[62] Valero M.A., Navarro M., Garcia-Bodelon M.A., Marcilla A., Morales M., Garcia J.E., Hernandez J.L., Mas-Coma S. (2006). High risk of bacterobilia in advanced experimental chronic fasciolosis. Acta Trop..

[63] Valero M.A., Girones N., Garcia-Bodelon M.A., Periago M.V., Chico-Calero I., Khoubbane M., Fresno M., Mas-Coma S. (2008). Anaemia in advanced chronic fasciolosis. Acta Trop..

[64] Valero M.A., Bargues M.D., Khoubbane M., Artigas P., Quesada C., Berinde L., Ubeira F.M., Mezo M., Hernandez J.L., Agramunt V.H. (2016). Higher physiopathogenicity by *Fasciola gigantica* than by the genetically close *F. hepatica*: Experimental long-term follow-up of biochemical markers. Trans. R. Soc. Trop. Med. Hyg..

[65] Mas-Coma S., Bargues M.D., Valero M.A. (2014). Diagnosis of human fascioliasis by stool and blood techniques: Update for the present global scenario. Parasitology.

[66] Zumaquero-Rios J.L., Sarracent-Perez J., Rojas-Garcia R., Rojas-Rivero L., Martinez-Tovilla Y., Valero M.A., Mas-Coma S. (2013). Fascioliasis and intestinal parasitoses affecting schoolchildren in Atlixco, Puebla State, Mexico: Epidemiology and treatment with nitazoxanide. PLoS Negl. Trop. Dis..

[67] World Health Organization (2006). WHO Consultation to Develop a Strategy to Estimate the Global Burden of Foodborne Diseases. Taking Stock and Charting the Way Forward.

[68] Valero M.A., Perez-Crespo I., Periago M.V., Khoubbane M., Mas-Coma S. (2009). Fluke egg characteristics for the diagnosis of human and animal fascioliasis by *Fasciola hepatica* and *F. gigantica*. Acta Trop..

[69] Curtale F., Hassanein Y.A.W., El Wakeel A., Barduagni P., Savioli L. (2003). The School Health Programme in Behera: An integrated helminth control programme at Governorate level in Egypt. Acta Trop..

[70] Farag H.F., Barakat R.M.R., Ragab M., Omar E. (1979). A focus of human fascioliasis in the Nile Delta, Egypt. J. Trop. Med. Hyg..

[71] Savioli L., Chitsulo L., Montresor A. (1999). New opportunities for the control of fascioliasis. Bull. WHO.

[72] Gandhi P., Schmitt E.K., Chen C.W., Samantray S., Venishetty V.K., Hughes D. (2019). Triclabendazole in the treatment of human fascioliasis: A review. Trans. R. Soc. Trop. Med. Hyg..

[73] Valero M.A., Panova M., Mas-Coma S. (2005). Phenotypic analysis of adults and eggs of *Fasciola hepatica* by computer image analysis system. J. Helminthol..

[74] Stephenson L.S. (1997). Appendix VI: Statistical treatments of egg count data. The Impact of Helminth Infection on Human Nutrition.

